# Supplementary material for: Development and validation of a multi-parameter nomogram for predicting prostate cancer: a retrospective analysis from Handan Central Hospital in China
Source: PeerJ. 2022 Mar 2;10:e12912. doi: 10.7717/peerj.12912 (PMC8898009; doi:10.7717/peerj.12912)
Supplement: Supplemental Information 3 [file peerj-10-12912-s003.docx]

continuous variables: Age, tPSA, fPSA, PV( prostate volume), FT(%fPSA), PSAD.

qualitative variables:

lable 1: prostate cancer lable 0: non-prostate cancer

DRE 1 :suspect cancer DRE 0 :normal

TRUS1:positive TRUS 0:negative

BMI 0:BMI≤22.9kg/m^2^

BMI 1:BMI23-27.4kg/m^2^

BMI 0:BMI≥27.5kg/m^2^

hp:Hypertension0: No Hypertension1: Yes

dia:Diabetes0: No Diabetes1: Yes
